# Case report: Progressive multifocal leukoencephalopathy co-occurring with neurosarcoidosis: early brain biopsy and appropriate therapy for PML resulted in a favorable prognosis

**DOI:** 10.3389/fimmu.2024.1447992

**Published:** 2024-10-11

**Authors:** Qiannan Wang, Shintaro Tsuboguchi, Kouichirou Okamoto, Mari Tada, Akiyoshi Kakita, Kazuo Nakamichi, Makoto Oishi, Masato Kanazawa, Osamu Onodera

**Affiliations:** ^1^ Department of Neurology, Brain Research Institute, Niigata University, Niigata, Japan; ^2^ Department of Translational Research, Brain Research Institute, Niigata University, Niigata, Japan; ^3^ Department of Pathology, Brain Research Institute, Niigata University, Niigata, Japan; ^4^ Department of Virology 1, National Institute of Infectious Diseases, Tokyo, Japan; ^5^ Department of Neurosurgery, Brain Research Institute, Niigata University, Niigata, Japan

**Keywords:** PML, neurosarcoidosis, brain biopsy, avoiding immunosuppressive therapy, favorable prognosis

## Abstract

Progressive multifocal leukoencephalopathy (PML) is a rare central nervous system disease caused by JC virus (JCV) infection. Human immunodeficiency virus (HIV) infection is the greatest risk factor for PML. Other immunological diseases, including systemic sarcoidosis, have also been reported as risk factors for PML. Herein, we report a case of PML co-occurring with neurosarcoidosis. Early diagnosis using brain biopsy and appropriate therapeutic interventions achieved favorable outcomes. PML in patients with active intracranial neurosarcoidosis is extremely rare. We believe that it is important to perform brain biopsy at an early stage to allow diagnosis, even for central nervous system involvement with a progressive parenchymal lesion in patients with sarcoidosis, if PML is possible.

## 1 Introduction

Progressive multifocal leukoencephalopathy (PML) is an opportunistic and rare central nervous system infection caused by infection with the JC virus (JCV). Human immunodeficiency virus (HIV) infection is the greatest risk factor for PML; however, other immunological diseases, including systemic sarcoidosis, have also been reported as risk factors (1). Herein, we report a case of PML co-occurring with neurosarcoidosis in a patient with systemic sarcoidosis. PML is extremely rare in patients with active intracranial neurosarcoidosis. Early diagnosis using brain biopsy and appropriate therapeutic interventions resulted in favorable outcomes.

## 2 Case description

### 2.1 History

**Figure 1:**
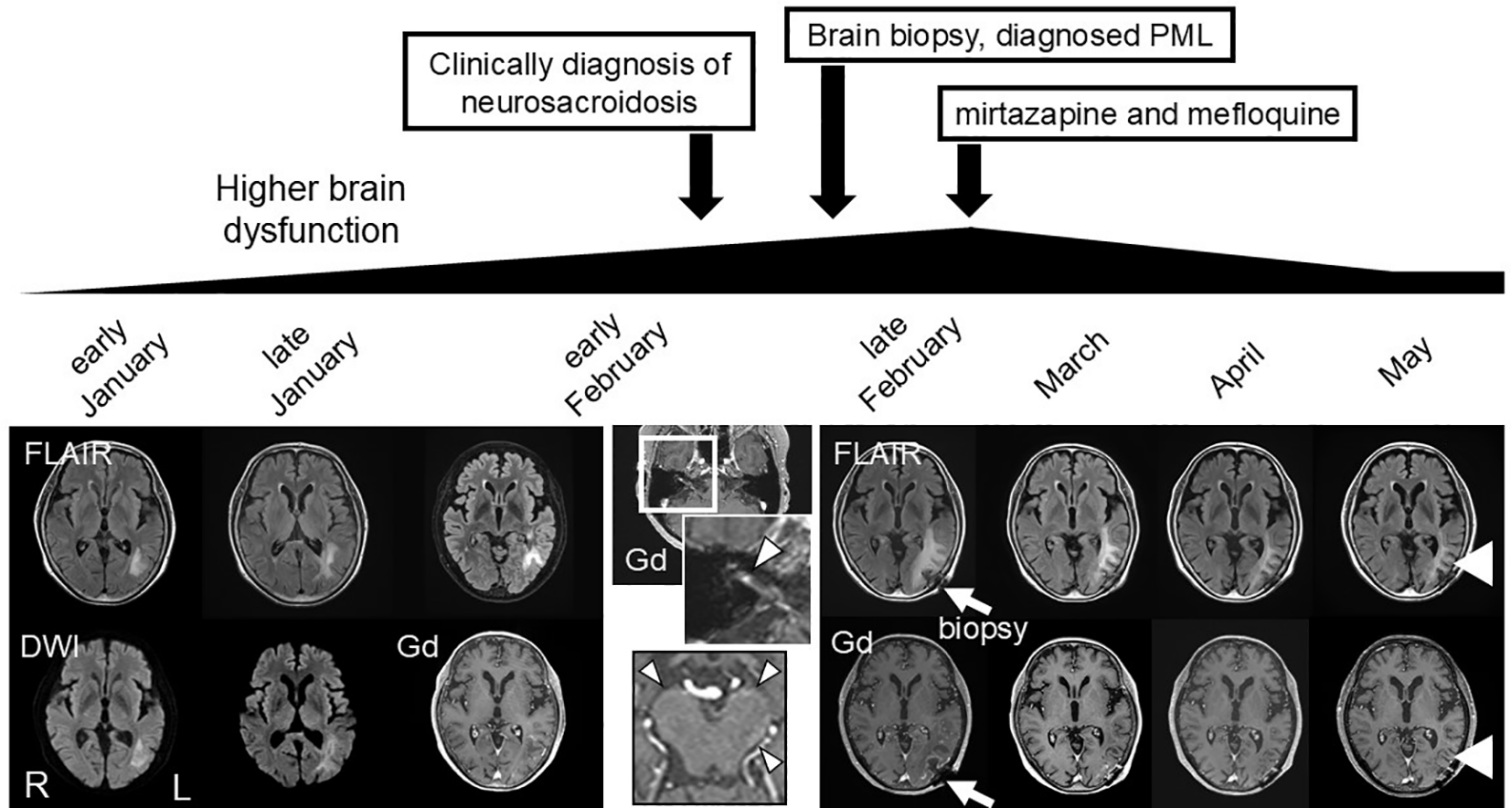
Clinical course and MRI findings. Magnetic resonance imaging (MRI) findings. Diffusion-weighted imaging (DWI) and fluid attenuated inversion recovery (FLAIR) revealed an enlarging hyperintense lesion in the left temporal, occipital, and parietal lobes before treatment. Gadolinium-enhanced T1-weighted images revealed an enhancing area in the lesion, and enhancement of the right cranial nerve VII (arrowhead), and leptomeninges in the brainstem (arrowhead). Brain biopsy was performed for the enhancing brain parenchymal lesion in late February (arrow), and mirtazapine and mefloquine treatment was started in March. Higher brain dysfunction and the MRI lesion gradually improved following the treatment (arrowhead).

A 65-year-old female presented with difficulties in speech, reading, and writing in November. She had medical histories of breast cancer and renal cancer 15 and 14 years earlier, respectively. She also had cutaneous, ocular, and pulmonary sarcoidosis 3 years before, which was diagnosed by skin biopsy. Due to the low disease activity of sarcoidosis, the patient did not receive immunosuppressive therapy. The patient was bothered by difficulties in speech, reading, and writing in January of following year. Brain magnetic resonance imaging (MRI) revealed a hyperintense lesion on diffusion-weighted imaging (DWI) and fluid-attenuated inversion recovery (FLAIR) imaging in the white matter of the left temporo-occipital region as depicted in **Figure 1**. The patient was initially suspected of cerebral infarction at a local hospital. However, her symptoms gradually worsened, and she was unable to understand conversations. The white matter lesion enlarged (**Figure 1**). In early February, the patient was referred to our hospital (Day 1).

### 2.2 Examination

Her general and physical findings at admission were normal. Neurological examination revealed higher brain dysfunction, disorientation, aphasia, right apraxia, alexia, agraphia, acalculia, and right-left disorientation. Laboratory examination revealed elevated soluble interleukin-2 receptor (sIL-2R) 2350 U/mL (normal range 122–496 U/mL), and angiotensin I-converting enzyme (ACE) 45.8 U/L (normal range 8.3–21.4 U/L). Hepatic, renal, and coagulation functions were normal. Cerebrospinal fluid (CSF) analysis yielded the following normal results: total cells, 4/mm^3^ (all mononuclear cells); albumin, 29 mg/dL; glucose, 52 mg/dL (blood glucose, 87 mg/dL); and CSF-ACE, 0.78 U/L. However, in the CSF, the values of protein, 59 mg/dL, β2-microglobulin 5.51 μg/mL, sIL-2R 195 U/mL, and the cluster of differentiation (CD) 4/8 ratio 10.4 were elevated. Oligoclonal bands were positive. CSF cultures were negative for both bacterial and acid-fast bacillus infections. Right facial nerve palsy developed on the second day of hospitalization. On day 3, gadolinium-enhanced MRI revealed enhancement of the leptomeninges at the brainstem and the right cranial nerve VII, characteristic MRI findings for neurosarcoidosis, with a left temporo-occipital parenchymal lesion (**Figure 1**).

## 3 Diagnostic assessment

**Figure 2:**
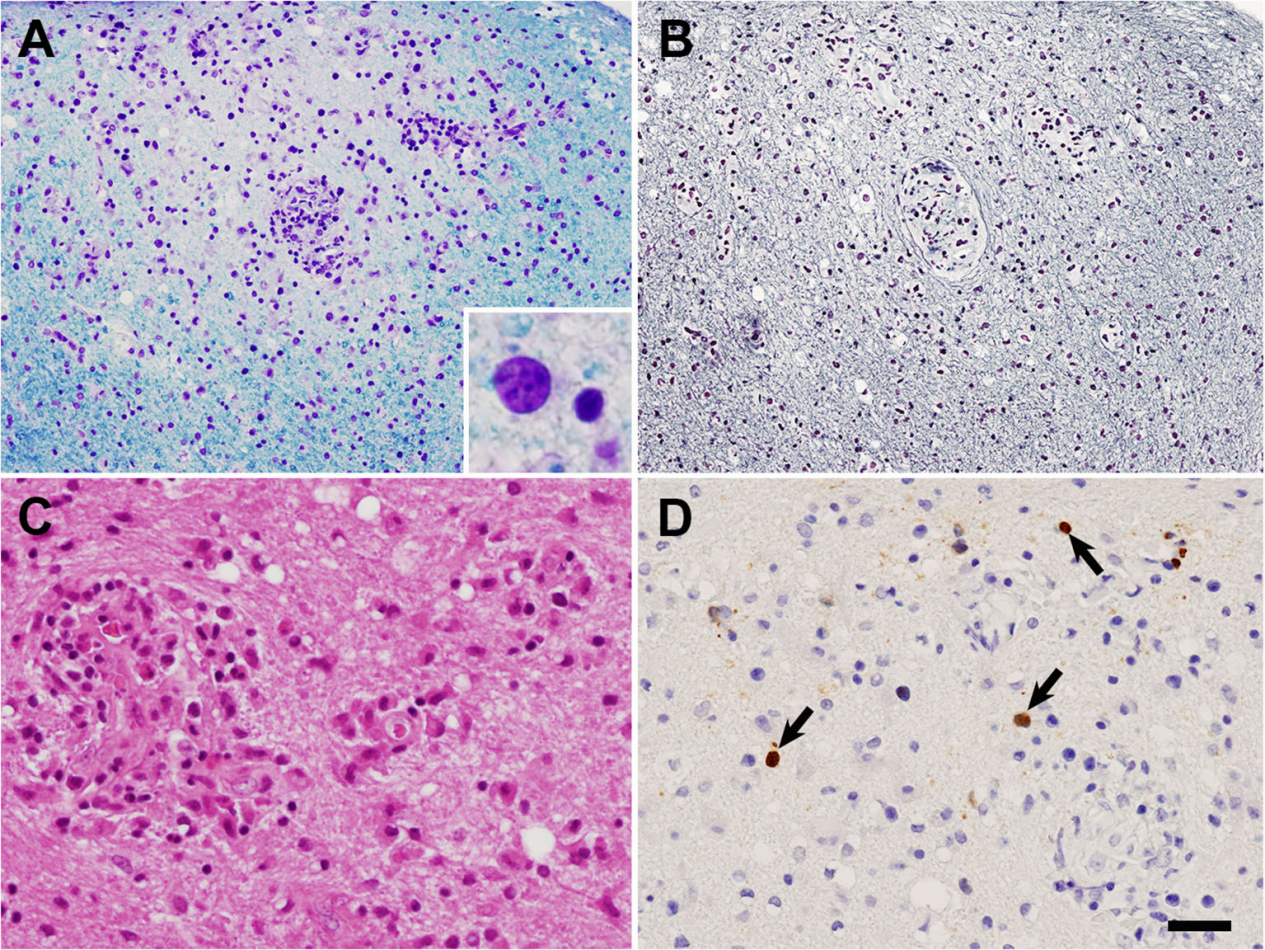
Histological findings of brain biopsy. **(A, B)** Adjacent sections of the white matter. A small ill-defined demyelinating lesion with well-preserved axons can be observed. Oligodendroglial enlarged nuclei [inset in **(A)**] were scattered mainly in the periphery of the lesion, on both **(A)**, Klüver-Barrera staining; and **(B)**, Bodian staining. **(C)** Reactive astrocytes and perivascular inflammatory cell infiltration in the demyelinating lesion on Hematoxylin-eosin staining. **(D)** Oligodendroglial JCV-positive nuclei (arrows) in the demyelinating lesion observed following JCV VP1 immunohistochemistry. Bar = 50 μm in **(A, B)**, 25 μm in **(C, D)**, 7 μm in inset in **(A)**.

Based on the MRI findings and results of the CSF analysis, neurosarcoidosis was strongly suggested initially. However, the white matter lesion rapidly extended to the right temporo-parieto-occipital lesion, which seemed atypical as a sarcoid lesion. Open biopsy was performed through craniotomy for the left temporo-occipital lesion to explore the possibility of lymphoma or PML (day 7). Histopathological examination revealed multiple small demyelinating foci with well-preserved white-matter axons (**Figure 2**). Oligodendroglial enlarged nuclei were scattered mainly in the periphery of the demyelinating foci, some of which were labelled with anti-JCV antibody. Although inflammatory cell infiltration was observed in the perivascular area, non-caseating granulomas, a characteristic histological feature of sarcoidosis, were not evident. Based on these findings, the diagnosis of PML was confirmed. Then mirtazapine and mefloquine were administered. One month later, PCR for JCV performed at National Institute of Infectious Diseases on the formalin-fixed paraffin-embedded (FFPE) brain tissue (1170 copies/cell) and CSF (205 copies/ml) revealed positive results, supporting the diagnosis of PML. After starting therapy for PML, the patient’s higher brain dysfunction and the MRI findings gradually improved (**Figure 1**). The patient was discharged in April, and her clinical course after discharge was uneventful. She could walk by herself, and could perform daily activities with her family’s support.

## 4 Discussion

Herein, we report a case of PML complicates sarcoidosis with CNS involvement. Avoiding inappropriate steroid therapy and treatment with mirtazapine and mefloquine for PML may have resulted in a favorable prognosis in this patient. Although HIV infection is the largest risk factor for PML (1), it is crucial to recognize that other immunosuppressive or immunological diseases, such as multiple sclerosis and systemic sarcoidosis, also increase the risk of PML. Systemic sarcoidosis is known to be complicated by PML, even in the absence of therapeutic immune suppression (2); however, PML complicates neurosarcoidosis has rarely been reported (3), so PML and neurosarcoidosis likely co-occurred. We initially diagnosed the patient with neurosarcoidosis and planned to administer steroids for following reasons: First, the patient presented with facial nerve palsy, and gadolinium-enhanced MRI revealed enhancement of the leptomeninges and the right cranial nerve VII (4). Second, CSF analysis revealed increased CSF-CD4/CD8 ratio. CSF-CD4/CD8 ratio >5 is highly suggestive of active neurosarcoidosis (5). These findings strongly suggested a clinical diagnosis of neurosarcoidosis, although this was not pathologically proven. However the extending white matter lesion was atypical as a neurosarcoid lesion, a brain biopsy was performed. Finally, the patient was diagnosed PML co-occurring with neurosarcoidosis. Steroid administration has been shown to worsen PML (6); therefore, we avoided steroid administration for neurosarcoidosis, and initiated PML therapy (7). While there is very little data on the effect of mirtazapine and mefloquine on the sarcoidosis-associated PML disease course (3), we administered them. Therefore, it is unclear whether the improvement in symptoms is due to the effectiveness of mirtazapine and mefloquine or occurs naturally. Anti-TNF-α therapy has been reported to be effective (8). In our case, anti-TNF-α therapy may have been a therapeutic candidate. Non-HIV-associated PML has a poor prognosis, with an overall median survival of only 3 months (9). However, in our patient, prioritizing PML therapy led to a favorable clinical course.

## 5 Conclusion

Brain biopsy is crucial for the accurate and rapid diagnosis of PML, even in systemic sarcoidosis with active CNS involvement. When PML coexists with neurosarcoidosis, prioritizing PML therapy and avoiding immunosuppressive therapy can lead to a favorable prognosis.

## Data Availability

The raw data supporting the conclusions of this article will be made available by the authors, without undue reservation.
